# Physical vs. Behavioral Clinical Signs in Horses with Squamous and Glandular Gastric Ulcers

**DOI:** 10.3390/vetsci13040328

**Published:** 2026-03-27

**Authors:** José Pimenta, Bruna Martins, Alexandre Mexedo, Rita Campilho, Filipe Silva, Mário Cotovio

**Affiliations:** 1 CIVG—Vasco da Gama Research Center, EUVG—Vasco da Gama University School, 3020-210 Coimbra, Portugal; aluno.1800855@euvg.pt; 2 CECAV—Veterinary and Animal Research Center, University of Trás-os-Montes e Alto Douro, 5000-801 Vila Real, Portugal; fsilva@utad.pt (F.S.); p4980@ulusofona.pt (M.C.); 3 Associate Laboratory for Animal and Veterinary Sciences (AL4AnimalS), 5000-801 Vila Real, Portugal; 4 Independent Researcher, 5000-801 Vila Real, Portugal; alexandrecgmexedo@gmail.com; 5 Veterinary Teaching Hospital of the University of Trás-os-Montes e Alto Douro (HVUTAD), 5000-801 Vila Real, Portugal; ritacampilhosousa@gmail.com; 6 Veterinary Sciences Department, University of Trás-os-Montes e Alto Douro, 5000-801 Vila Real, Portugal; 7 I-MVET, Faculty of Veterinary Medicine, Lusófona University, Campo Grande 376, 1749-024 Lisbon, Portugal

**Keywords:** equine, clinical signs, EGUS, ESGD, EGGD, gastroscopy, behavior

## Abstract

Equine Gastric Ulcer Syndrome is a highly prevalent condition, yet its clinical diagnosis remains challenging due to the nonspecific nature of many associated signs. The relationship between the anatomical location of gastric ulcers and clinical presentation is still poorly defined. This retrospective study aimed to evaluate the association between gastric ulcer location and the clinical signs observed in horses. Medical records of 52 horses diagnosed with gastric ulcers by gastroscopy were retrospectively reviewed. Clinical signs were classified as physical, behavioral, or mixed, and ulcer localization was categorized as Equine Squamous Gastric Disease (ESGD), Equine Glandular Gastric Disease (EGGD), or mixed. Associations between variables were analyzed using chi-square or Fisher’s exact tests, with *p* < 0.05 considered significant. A significant association was identified between the type of clinical signs and ulcer location (*p* < 0.001). Physical clinical signs were predominantly associated with ESGD, whereas behavioral and mixed signs were more frequently observed in horses with EGGD or mixed ulceration. Furthermore, both the type of clinical signs and ulcer location were associated with the horse’s sport activity. No association was found between ulcer severity and clinical signs nor between ulcer location and sex. These findings suggest that gastric ulcer location influences clinical presentation, indicating that ESGD and EGGD may be associated with different clinical sign patterns.

## Simple Summary

Equine Gastric Ulcer Syndrome is frequently diagnosed, yet recognition of the disease can be challenging. Some symptoms can be unspecific, and different horses do not necessarily present with the same clinical signs. In this retrospective study, clinical records from 52 horses with gastric ulcers enhanced by gastroscopy were reviewed to explore whether the location of the ulcers was associated with the type of clinical signs observed. Horses with ulcers affecting the squamous region of the stomach exhibited physical signs more frequently, whereas horses with glandular or mixed ulcers more commonly presented behavioral or combined signs. Additionally, associations were observed between the type of clinical signs and the horse’s sport activity and between sport activity and ulcer location. Ulcer severity and sex were not associated with clinical presentation These results indicate that the location of gastric ulcers may influence how the disease manifests clinically, which could help clinicians better interpret clinical signs when gastric ulcer disease is suspected.

## 1. Introduction

Equine Gastric Ulcer Syndrome (EGUS) is widely recognized as one of the most common disorders affecting the equine stomach and represents a significant concern for equine health, welfare, and athletic performance worldwide [1,2]. Gastroscopic and post-mortem studies have consistently reported a high prevalence of gastric ulceration across a broad range of equine populations, particularly among horses involved in athletic activity [3,4,5]. Despite its high prevalence, EGUS remains challenging to diagnose clinically, as affected horses often exhibit nonspecific clinical signs that may be overlooked by owners or by clinicians who are less attuned to subtle changes in behavior or performance [1,6,7,8].

The terminology EGUS encompasses two distinct disease entities that differ in anatomical location and underlying pathophysiology: Equine Squamous Gastric Disease (ESGD) and Equine Glandular Gastric Disease (EGGD) [2]. ESGD affects the non-glandular squamous mucosa of the stomach and is primarily associated with acid-related injury. In contrast, EGGD involves the glandular mucosa, most commonly the pyloric and antral regions, and is thought to arise from the impairment of normal mucosal defense mechanisms. These differences suggest that ESGD and EGGD represent biologically distinct conditions rather than variations of the same disease process [2,9]. However, ESGD and EGGD can occur simultaneously in the same horse.

A wide range of clinical signs has been attributed to EGUS in both clinical practice and the scientific literature. Commonly reported signs include recurrent or mild colic, changes in appetite or eating behavior, loss of body condition, reduced performance, alterations in coat quality, diarrhea, and a variety of behavioral changes such as resistance during riding, girth aversion, irritability, nervousness, or the development of stereotypic behaviors [4,10,11,12,13,14,15,16,17]. However, none of these signs are specific to gastric disease, and considerable overlap exists with other gastrointestinal, musculoskeletal, or systemic conditions [2].

The relationship between clinical signs and the presence of gastric ulceration remains inconsistent. While some studies have identified associations between certain clinical signs and EGUS, others have failed, particularly when relying on owner-reported observations [6,7,8]. Moreover, several investigations have shown that horses with confirmed gastric ulceration may exhibit minimal or no clinical signs, whereas horses presenting with suggestive signs do not always show clinical improvement following the resolution of gastric lesions [2,6,7,8]. Although these findings highlight the limited reliability of clinical signs alone, symptomatology continues to represent a central component of the diagnostic process, as they often constitute the primary reason for gastroscopic investigation [7,8,18,19,20,21]. Therefore, a better understanding of how clinical signs relate to gastric ulcer characteristics may improve their diagnostic value.

According to the European College of Equine Internal Medicine Consensus Statement regarding Equine Gastric Ulcer Syndrome in adult horses, “differences *in clinical signs between ESGD versus EGGD are unknow but warrant investigation*” [2]. Despite that, only limited attention has been given to whether the location of gastric ulceration influences the type of clinical signs observed and available evidence remains sparse and sometimes contradictory. As a result, it is still unclear whether squamous, glandular, or mixed gastric ulceration is associated with distinct clinical presentations.

The aim of the present study was to retrospectively evaluate the relationship between the anatomical location of gastric ulcers (ESGD, EGGD, and mixed lesions) and the clinical signs observed in horses undergoing gastroscopic examinations.

## 2. Materials and Methods

### 2.1. Data Collection

Sixty-nine medical records along with digital endoscopy files of horses diagnosed with gastric ulcers by gastroscopy between 2014 and 2025 were retrospectively analyzed. Some cases were managed at the Veterinary Teaching Hospital of the University of Trás-os-Montes e Alto Douro (HVUTAD), whereas others were followed during private clinical practice. Not all medical records were complete; therefore, some clinical data was unavailable for certain horses. This study was approved by the Institutional Ethics Committee of Vasco da Gama University School (31/2025, 6 January 2026).

### 2.2. Inclusion and Exclusion Criteria

The study sample was restricted to horses in which gastric ulceration was the only diagnosed condition. Regarding ESGD, horses were assessed using the grading system described in the Equine Gastric Ulcer Syndrome Consensus [2], and all lesion grades within this classification were considered eligible for inclusion, including grade 1 lesions.

Horses presenting with concurrent musculoskeletal, neurologic, or other gastrointestinal diseases capable of explaining the clinical signs, as well as horses signalized for management inadequacies that could account for these signs (e.g., erroneous or recent changes in dietary/feeding, housing, or training practices) were excluded. Horses with a history of receiving any anti-ulcer medication (e.g., sucralfate, misoprostol, omeprazole, and H2 antagonists) within four weeks prior to the gastroscopy exam were also excluded. Cases were additionally excluded when the reported clinical signs had not been clearly confirmed by the attending veterinarian or when clinical signs were not explicitly documented in the medical record or were considered uncertain.

### 2.3. Medical Records Information

Clinical data included sex; age; horse’s use/sport activity (leisure, dressage, jumping, and racing); clinical signs prior to gastroscopy; gastroscopic localization of gastric ulcers (ESGD; EGGD; and mixed); and severity/degree of ESGD classified according to Equine Gastric Ulcer Syndrome Consensus [2] (Table 1). Glandular gastric ulcers were classified dichotomously as present or absent.

### 2.4. Clinical Signs

Clinical signs were divided into three categories: physical; behavioral; and mixed (according to Table 2).

Physical clinical signs were defined as health-related findings that can be identified during the general clinical examination.

Behavioral clinical signs were defined as deviations from the horse’s normal behavior based on owner reports and confirmed by the veterinarian. Horses with behavioral signs exclusively had no detectable abnormalities on general physical examination.

Regarding physical clinical signs, recurrent colic was defined as the occurrence of more than one episode of colic within a two-week period, with spontaneous resolution and no requirement for medical or surgical veterinary treatment. Loss of body condition encompassed failure to gain weight despite adequate nutritional and training management and in the absence of an identifiable cause. Loss of body condition was assessed by the attending veterinarian and defined as a body condition score ≤5 according to the Henneke scale [22]. Poor coat condition was characterized by the presence of a lackluster or rough hair coat in horses with no recent changes in diet. Diarrhea was defined as the occurrence of multiple episodes, consecutive or intermittent, of feces softer than normal in the absence of dietary or management changes.

Concerning behavioral signs, nervousness or irritability was defined as a change in behavior in multiple daily situations, such as in the stall, paddock, or during transport, considering the horse’s previous behavior. Poor performance referred to results or abilities below the horse’s normal or expected level, sometimes encompassing exercise intolerance. Girthiness/girth aversion was considered present when horses showed abnormal behaviors during saddling or girth tightening, including ear pinning, biting, tail swishing, moving away, or pawing. Negative behavior during riding was classified in horses exhibiting aggressive responses to the rider’s aids or showing aggressive reactions while mounted, including rearing, tail swishing, fleeing, refusing specific exercises, kicking, and bucking. Inappetence was classified in horses presenting picky or selective eating behavior, as well as in cases of partial anorexia. Stereotypic behaviors referred to repetitive, purposeless actions.

### 2.5. Statistical Analysis

Data normality was assessed using the Shapiro–Wilk test. Associations between categorical variables were evaluated using the chi-square test or Fisher’s exact test, as appropriate. Differences in median values between variables with two or three categories were assessed using the Mann–Whitney U test and the Kruskal–Wallis test, respectively. Spearman correlation test was also performed. All statistical tests were considered statistically significant at *p* < 0.05. Statistical analyses were performed using Jamovi statistical software (version 2.3.28).

## 3. Results

### 3.1. Sample Characterization

Out of the 69 medical records, this study included 52 case records of horses (23 from HVUTAD and 29 from private clinical practice) with a mean age of 11 ± 3.18 years (range: 4–20 years). Of these, 28 were males (including seven geldings) and 24 were females. Nineteen horses were used for racing, 17 for show jumping, nine for dressage, and seven for leisure riding.

### 3.2. Gastroscopy Findings

Regarding the location of gastric ulcers, 22 horses had ulcers affecting the squamous mucosa, 18 had mixed lesions involving both the squamous and glandular regions, and 12 had ulcers confined to the glandular mucosa.

With respect to the severity of ESGD, nine horses had grade 1 lesions, 15 had grade 2, 12 had grade 3, and four had grade 4.

### 3.3. Clinical Signs

Regarding the clinical signs observed, 15 horses exhibited physical signs (28.8%), 28 exhibited behavioral signs (53.8%), and nine showed mixed clinical signs (17.3%). Some horses presented more than one physical or behavioral sign simultaneously.

Among physical clinical signs, the loss of body condition was observed in 15 horses, recurrent colic in seven, poor coat condition in five, and diarrhea in one horse. With respect to clinical behavioral signs, 18 horses showed negative behavior during riding, 17 exhibited girthiness, six had inappetence, five showed poor performance, five demonstrated nervousness or irritability, and one horse displayed stereotypic behavior (crib biting).

### 3.4. Association Between Clinical Signs and Gastroscopic Findings

A statistically significant association was identified between the type of clinical signs and gastric ulcer localization (*p* < 0.001) (Table 3). Physical clinical signs were predominantly associated with ESGD, with 13 of 15 horses presenting physical signs showing lesions confined to the squamous mucosa. In contrast, behavioral and mixed clinical signs were more frequently observed in horses with EGGD or mixed gastric ulceration. Notably, no horses with glandular ulcers exclusively exhibited physical clinical signs.

The severity/score of squamous gastric ulcers showed no significant influence on the type of clinical signs observed (*p* = 0.93).

### 3.5. Relationship Between Clinical Variables and Gastroscopic Findings

No significant differences were observed in ESGD scores among horses with different sport activities (racing, show jumping, dressage, or leisure riding) (*p* = 0.17). However, when the ESGD score was divided into two groups (Group 1—horses with only grade 1 lesions; Group 2—horses with grade 2, 3, or 4 lesions), a significant association was observed between lesion severity and discipline (*p* = 0.01). Horses performing racing had no lesions with score 1. No association was found between ESGD score (grouped into grade 1 lesions and grades 2–4 lesions) and the type of clinical signs (*p* = 1.0) or the number of clinical signs reported (*p* = 0.12).

There was an association between the type of clinical signs presented (physical, behavior, or mixed) and the horse’s use/sport activity (*p* = 0.02). Racing horses predominantly exhibited behavioral or mixed clinical signs, whereas jumping horses mainly presented physical or behavior signs (Table 4). Furthermore, an association was found between the horse’s sport activities and gastric ulcer localization (*p* = 0.03), indicating that horses used for racing were more frequently affected by glandular or mixed lesions (Table 5).

Sex did not present any kind of statistically significant association with gastric ulcer localization (*p* = 0.5); ESGD scores (*p* = 0.96); and neither with the type of clinical signs observed (*p* = 0.08). Although females more frequently exhibited behavioral clinical signs, this trend did not reach statistical significance.

Age had no significant influence on either the type of clinical signs (*p* = 0.16) or gastric ulcer localization (*p* = 0.84). No correlation was found between age and the ESGD severity score (*p* = 0.4).

## 4. Discussion

The present study evaluated the association between the anatomical location of gastric ulceration and the type of clinical signs observed in horses. Although Equine Gastric Ulcer Syndrome has been extensively studied in terms of prevalence, risk factors, and treatment, limited attention has been given to potential differences in clinical presentation between Equine Squamous Gastric Disease and Equine Glandular Gastric Disease [18,23]. Most published studies describe clinical signs in a descriptive manner or focus on their diagnostic value for the presence of gastric disease rather than statistically assessing their relationship with ulcer location [6,7,8,11,24,25,26,27]. Consequently, available evidence addressing whether ESGD and EGGD are associated with distinct patterns of clinical expression remains scarce.

With respect to clinical signs, which represent the focus of the present study, the available literature remains highly inconsistent. Before comparing our findings with published reports, it is important to acknowledge that the present study adopted a methodological approach that differs from that of most previous investigations, which limits the direct and detailed comparisons of results. In this study, clinical signs were not evaluated individually but were instead grouped into three categories: physical, behavioral, and mixed. This strategy was selected because many horses with EGUS do not present with a single isolated clinical sign; consequently, assessing individual signs in isolation may be less representative of clinical reality and may also reduce the applicability of inferential statistical analyses. Furthermore, although this does not preclude comparison with other studies, all clinical signs were recorded and confirmed by a veterinarian rather than relying solely on owner-reported observations, as in many previously published studies, thereby increasing confidence in the accuracy of the collected data.

Despite the substantial lack of consensus regarding the clinical presentation of horses with ESGD and EGGD, some published studies are consistent with our findings. In general, horses with EGGD appear more prone to behavioral abnormalities [7,23,25]. Several authors have reported an association between glandular lesions and poor performance, as well as reduced behavioral compliance during riding, and stress has been proposed as one of the main mechanisms involved in the pathophysiology of EGGD [16,17,23]. Girthiness is among the most frequently reported clinical signs in the literature of EGUS; however, no consistent association has been established between this sign and either ESGD or EGGD, although some studies have described a higher incidence in horses with EGGD [6,7,10,11,12,13,17,26,28,29]. Regarding squamous lesions, previous studies have reported associations with loss of body condition and colic, which is also in agreement with the results of the present study [4,15,18,23,27]. Nevertheless, some studies have identified associations between various behavioral problems and ESGD, further highlighting the need for additional investigations [18,25,27,30]. The association between ESGD severity and clinical expression remains unclear. While one study observed a significant influence of ESGD severity on behavioral signs, other studies failed to detect differences in clinical presentation across severity grades, indicating that horses with ESGD may exhibit similar signs regardless of lesion severity [6,10,11].

Although a statistically significant difference in clinical signs presentation was detected between horses affected by ESGD and those affected by EGGD, which may represent a novel finding not previously reported to our knowledge, further studies using a similar methodological approach and larger sample sizes are required to confirm or refute these results. As highlighted in a recent review, when interpreting clinical signs in horses with gastric ulcer disease, it is essential to distinguish between these two disease entities to better understand potential differences in their clinical expression [9].

Despite the significant association identified between the sport activity and clinical sign category, this result should be interpreted with caution, as it may reflect a sample-specific effect driven by the limited number of horses per sport category. In the authors’ clinical experience, dressage horses with EGUS commonly present pronounced behavioral disturbances; however, the limited sample size likely precluded the detection of this pattern in the present study. Similarly, the association observed between discipline and ESGD score, when divided into two groups, should be interpreted with caution. Although the higher intensity of training may predispose racing horses to higher ESGD scores, the low number of horses with ESGD grade 1 included in this study (n = 9) does not allow strong conclusions to be drawn.

In contrast, the association between athletic activity and gastric ulcer localization has been widely investigated. In the present sample, racing horses showed a higher proportion of glandular and mixed ulcers, which does not fully agree with previous reports [18,19,20,27,31]. Some studies have described a greater prevalence of squamous ulcers in racing horses, mainly attributed to increased abdominal compression and gastric contraction during canter, which favors acid splashing and exposure of the non-glandular mucosa to gastric acid [19,20,27,32]. Nevertheless, no clear consensus has been reached, and this relationship is thought to be strongly influenced by additional factors such as exercise intensity and frequency, transport, and competition [9,12,32]. As these variables were not available in the present study, further detailed analysis was not possible.

Most horses included in this study were diagnosed with ESGD, followed by mixed ulcers and EGGD. According to the literature, the reported prevalence of ESGD and EGGD varies widely among studies, as it is influenced by several factors, including the characteristics of the sampled population, exercise regimen (e.g., intensity and frequency of training), management practices, and other husbandry-related variables [13,23,30,31,33]. Nevertheless, in addition to being more extensively documented, ESGD appears to have a higher overall prevalence in several published studies [13,18,21,23,30,31,33,34].

No association was found between age and ESGD severity in the present study, in contrast to some reports suggesting that ulcer severity increases with age [35]. Sex was also not associated with any of the variables evaluated, although other studies indicate that geldings have a higher risk of developing gastric ulcers [25,34,35]. However, because this study did not include a gastric ulcer-free control group, such risk-based analyses could not be performed.

Several limitations of this study must be acknowledged. The retrospective nature of the study relies on the accuracy and completeness of medical records, which was conducive to the lack of some clinical information. In addition, the relatively small sample size and the absence of a control group (horses without gastric ulcers) may limit the generalizability of the findings. The exclusion of horses with concurrent diseases was intended to reduce confounding but may also restrict applicability to more complex clinical cases commonly encountered in practice. Furthermore, glandular lesions were classified dichotomously as present or absent, which does not account for potential variation in severity or lesion subtype within the glandular mucosa. Additionally, although a clear association was identified in this work between ulcer location and the type of clinical signs, the biological mechanisms underlying this relationship remain unclear.

Despite these limitations, the present study provides clinically relevant evidence that ulcer location may influence the pattern of clinical signs. These findings may assist clinicians in interpreting presented complaints and in refining clinical suspicion of ESGD versus EGGD prior to gastroscopy.

## 5. Conclusions

This study demonstrated a significant association between gastric ulcer location and the type of clinical signs observed in horses. Squamous lesions were more often associated with physical signs, whereas glandular or mixed ulceration was more frequently linked to behavioral or mixed clinical presentations. These results suggest that, while clinical signs alone are poor predictors of gastric ulcer disease, ulcer location may influence the pattern of clinical expression.

Further studies are needed to better define the clinical significance of this relationship and to assess whether it can meaningfully support clinical suspicion and diagnostic decision-making. Overall, these findings provide a novel perspective on EGUS, contributing to a better understanding of its clinical manifestations and their possible association with lesion location.

## Figures and Tables

**Table 1:** Grading system for ESGD according to Equine Gastric Ulcer Syndrome Consensus.

Grade	Characterization of Lesions
0	Epithelium is intact, no appearance of hyperkeratosis
1	Intact mucosa, presence of areas of hyperkeratosis
2	Small, single, or multifocal lesions
3	Large, single, or extensive superficial lesions
4	Extensive lesions with areas of apparent deep ulceration

**Table 2:** Distribution of clinical signs between categories: physical, behavioral, and mixed.

Physical	Behavioral	Mixed
Recurrent colic	Nervousness/irritability	When both physical and behavioral clinical signs were presented
Loss of body condition	Poor performance	
Poor coat condition	Girthiness/girth aversion	
Diarrhea	Negative behavior during riding	
	Stereotypic behavior	
	Inappetence	

**Table 3:** Association between the type of clinical signs (physical; behavioral; or mixed) and gastric ulcers localization (ESGD; EGGD; or mixed).

	Clinical Signs				
Gastric Ulcer Localization	Physical	Behavioral	Mixed	Total	*p*
**ESGD**	13	9	0	22	
**EGGD**	0	9	3	12	0.001
**Mixed**	2	10	6	18	
**Total**	15	28	9	52	

**Table 4:** Association between the type of clinical signs (physical; behavioral; or mixed) and sport activities (racing, show jumping, dressage, or leisure).

	Clinical Signs				
Discipline	Physical	Behavioral	Mixed	Total	*p*
Racing	1	11	7	19	
Show Jumping	7	9	1	17	
Dressage	5	4	0	9	0.02
Leisure	2	4	1	7	
Total	15	28	9	52	

**Table 5:** Association between gastric ulcers localization (ESGD; EGGD; or mixed) and disciplines (racing, show jumping, dressage, or leisure).

	Gastric Ulcer Localization				
Discipline	ESGD	EGGD	Mixed	Total	*p*
Racing	3	8	8	19	
Show jumping	8	2	7	17	
Dressage	6	2	1	9	0.03
Leisure	5	0	2	7	
Total	22	12	18	52	

## Data Availability

The original contributions presented in this study are included in the article. Further inquiries can be directed to the corresponding author.
